# Disparities in quality of life among patients with breast cancer based on surgical methods: a cross-sectional prospective study

**DOI:** 10.1038/s41598-024-62105-z

**Published:** 2024-05-18

**Authors:** Yi Wang, Yibo He, Shiyan Wu, Shangnao Xie

**Affiliations:** Division of Breast Surgery, Department of Surgical Oncology, Hangzhou Cancer Hospital, Zhejiang, China

**Keywords:** Operable breast cancer, Oncoplastic surgery, Quality of life, Breast cancer, Quality of life

## Abstract

To determine the impact of breast conservation on quality of life and identify treatment-related and other demographic factors associated with post-breast cancer treatment quality of life. A prospective study was conducted on 392 women who underwent breast cancer surgery at Hangzhou Cancer Hospital from January 1, 2013, to December 31, 2022. Operable breast cancer patients who had completed all treatments except endocrine therapy were included. Patients with tumor recurrence/metastasis, bilateral or male breast cancer, and other primary malignancies were excluded. After enrollment, patients were asked to complete the BREAST-Q scale, and their pathological and medical records were reviewed. Analysis of variance was used to compare the quality of life scores among the groups. Univariate and multivariate linear regression analyses were performed to identify independent factors associated with quality of life scores in different domains. Participants completed the BREAST-Q scale at a median of 4.6 years after surgery. Quality of life scores varied based on the therapeutic strategy. Breast conservation has significant advantages over mastectomy in terms of breast satisfaction, psychosocial, and sexual well-being. Compared to oncoplastic breast-conserving surgery, mastectomy was independently associated with decreased breast satisfaction, psychosocial, and sexual well-being, while conventional breast-conserving surgery showed comparable outcomes to oncoplastic breast-conserving surgery in terms of these factors. Breast conservation leads to an improvement in quality of life compared to mastectomy. Oncoplastic breast-conserving surgery does not lead to a decrease in quality of life compared to conventional breast-conserving surgery and offers better outcomes compared to mastectomy.

## Introduction

Breast cancer is a prevalent global malignancy^1^, and breast-conserving surgery (BCS) with adjuvant radiotherapy (RT) is a well-established treatment for early-stage breast cancer^2,3^. However, up to 30% of BCS recipients express dissatisfaction with their postoperative appearance, necessitating corrective interventions^4^. In the 1980s, European surgeons introduced "oncoplastic breast-conserving surgery" (OBCS), which incorporates plastic surgery techniques for post-BCS breast defect reconstruction^5^.

While OBCS offers satisfactory long-term oncological results and broadens treatment possibilities for patients who would typically undergo mastectomies^6^, it involves more extensive incisions, additional tissue manipulation, and potential flap reconstruction in comparison to conventional breast-conserving surgery (cBCS)^7,8^. The procedures involved in OBCS are more complex, time-consuming, and costly. Given these complexities, is it still worthwhile to pursue breast conservation by OBCS? Some researchers have proposed whether the use of OBCS should be reduced^9^.

Understanding the impact on the quality of life of breast cancer survivors is crucial given its significant influence on medical decision-making^10,11^. Despite the widespread utilization of OBCS to conserve the breast and enhance its aesthetics, research on its impact on quality of life is limited and complicated due to the variability of surgical approaches. Consequently, this study aimed to assess the effect of breast conservation by OBCS on the quality of life of patients with operable breast cancer treated at Hangzhou Cancer Hospital from January 1, 2013, to December 31, 2022, and to elucidate the treatment and demographic factors associated with postoperative quality of life.

## Methods

### Materials and methods

This prospective, cross-sectional, case–control study was conducted at a single center. The inclusion criteria were operable breast cancer patients treated at Hangzhou Cancer Hospital between January 1, 2013, and December 31, 2022, who had completed all treatments except endocrine therapy and provided participation consent. The exclusion criteria were patients with tumor recurrence/metastasis, bilateral or male breast cancer, or other primary malignancies. Participants were categorized into two groups: BCS group (cBCS with RT subgroup and OBCS with RT subgroup), and unilateral MAST group (MAST with RT subgroup and MAST without RT subgroup). This study utilized the BREAST-Q scale^12^, which includes separate modules for BCS and MAST without reconstruction. The BCS module was used for the OBCS with RT subgroup because OBCS in this study predominantly referred to oncoplastic lumpectomy/glandular remodeling. BREAST-Q assesses six distinct domains: satisfaction with breasts, psychosocial well-being, physical well-being, sexual well-being, satisfaction with overall outcome, and satisfaction with care. Due to the elapsed time between surgery and questionnaire completion in this study, the domains of satisfaction with the overall outcome and satisfaction with care were excluded. Each domain was scored on a scale from 0 to 100, with higher scores indicating an enhanced quality of life. Differences in BREAST-Q scores were categorized as small (2–3 points), moderate (4–7 points), and large (8–10 points)^13^. Patient characteristics, collected using the questionnaire, included employment status, educational level, marital status, and economic status. Patients’ medical and pathological records were reviewed to determine the disease tumor, node, and metastasis (TNM) staging^14^, erythroblastic oncogene B (ERBB2; formerly HER2/neu or HER2) status, hormone receptor status, and body mass index (BMI). Information on surgery, chemotherapy (yes/no), RT, and endocrine therapy (yes/no) was obtained using a questionnaire in conjunction with medical records. The lymphedema status (yes/no) was assessed using the questionnaire's question regarding arm swelling. This study was approved by the Ethics Committee of Hangzhou Cancer Hospital, and all participants provided written informed consent. The study was performed in accordance with the Declaration of Helsinki and followed the guidelines of the International Society for Pharmacoeconomics and Outcomes Research (ISPOR) reporting guidelines.

### Statistical analysis

The experimental data were statistically analyzed using SPSS (version 29.0) software, and categorical covariates were expressed as numbers (percentages). Analysis of variance (ANOVA) was used to compare quality of life scores among the different groups. Univariate and multivariate linear regression analyses were used to determine the independent factors associated with the quality of life scores in each domain. Variables with two-tailed *P* ≤ 0.15 in the univariate analysis were included in the multivariate analysis model using a stepwise method to establish the final multivariate model. Differences with *P* < 0.05 were considered statistically significant.

### Ethics approval and consent to participate

This study was reviewed and approved by the ethics committee of Hangzhou Cancer Hospital (approval number: [hzch-2023] HS no.007). Written informed consent was obtained from every patient.

## Results

### Patient enrollment

After screening, 623 eligible patients were invited, 456 provided written informed consent and completed the survey, but three were found to not meet the inclusion criteria after enrollment. After excluding 61 participants who only completed a brief questionnaire, a total of 392 patients’ data were included in the statistical analysis.

### Patient, disease, and treatment characteristics

The interval between surgery and scale completion averaged 4.6 years (range: 0.33 to 9.83 years). Patient characteristics are detailed in Table 1. Majority were married, employed, had moderate economic status (income ¥30,000–200,000 per year), and high school or higher education. At surgery, 324 (82.7%) patients had a body mass index (BMI; calculated as weight in kilograms divided by height in meters squared) within the normal range (18.5 to 23.9 kg/m^2^), and 56 (14.3%) patients had a BMI of 24 kg/m^2^ or above. Among the patients, 39 (9.9%) had stage 0 breast cancer, 154 (39.3%) had stage I breast cancer, 158 (40.3%) had stage II breast cancer, and 41 (10.5%) had stage III breast cancer. The lesions on imaging before surgery of 253 (64.5%) patients measured two centimeters or less, 134 (34.2%) two to five centimeters, and 5 (1.3%) more than five centimeters. Chemotherapy was administered to 293 (74.7%) patients, with 121(30.9%) receiving neoadjuvant chemotherapy, and 273 (69.6%) patients received hormone therapy.

**Table 1:** Patient, disease, and treatment characteristics.

Characteristics	cBCS with RT (n = 51)	OBCS with RT (n = 88)	MAST with RT (n = 100)	MAST without RT (n = 153)	No. (%) (n = 392)
Age, y					
< 35	4	8	6	4	22 (5.6)
35–60	38	64	82	120	304 (77.6)
> 60	9	16	12	29	66 (16.8)
Baseline BMI, kg/m^2^					
< 18.5	1	4	4	3	12 (3.1)
18.5–23.9	39	72	82	131	324 (82.7)
≥ 24	11	12	14	19	56 (14.3)
Marital status					
Married	44	72	76	127	319 (81.4)
Single, divorced or widowed	4	11	17	16	48 (12.2)
Unknown	3	5	7	10	25 (6.4)
Income, ¥					
≥ 200,000	10	15	16	30	71 (18.1)
30,000–200,000	34	60	64	92	250 (63.8)
< 30,000	6	9	13	17	45 (11.5)
Unknown	1	4	7	14	26 (6.6)
Work status					
Work full time	36	68	80	118	302 (77.0)
Other	14	16	15	27	72 (18.4)
Unknown	1	4	5	8	18 (4.6)
Educational level					
High school or more	31	53	55	92	231 (58.9)
Other	19	31	39	58	147 (37.5)
Unknown	1	4	6	3	14 (3.6)
Tumor lesion on imaging before surgery, cm					
≤ 2	44	56	92	61	253 (64.5)
2–5	7	31	58	38	134 (34.2)
> 5	0	1	3	1	5 (1.3)
AJCC stage					
0	12	7	0	20	39 (9.9)
I	25	37	0	92	154 (39.3)
II	10	37	70	41	158 (40.3)
III	4	7	30	0	41 (10.5)
Chemotherapy					
Neoadjuvant	11	27	63	20	121 (30.9)
Adjuvant	21	38	27	86	172 (43.9)
No	19	23	10	47	99 (25.3)
Hormone therapy					
Yes	34	64	68	107	273 (69.6)
No	17	24	32	46	119 (30.4)
Time since surgery, y					
< 1	4	14	6	13	37 (9.4)
1–5	25	52	44	66	187 (47.7)
≥ 5	22	22	50	74	168 (42.9)
Nodal surgery					
Sentinel node biopsy only	40	62	0	153	255 (65.1)
Axillary dissection	11	26	100	0	137 (34.9)
Lymphedema					
Yes	8	14	22	17	61 (15.6)
No	43	74	78	136	331 (84.4)

BMI: body mass index; cm: centimeter; RT: radiotherapy; cBCS: conventional breast-conserving surgery; OBCS: oncoplastic breast-conserving surgery; MAST: mastectomy.

Treatment details including surgery, RT, and lymphedema are presented in Table 1. Among the patients, 88 (22.4%) underwent OBCS, 51 (13.0%) underwent cBCS, and 253 (64.5%) underwent unilateral MAST, among which 100 (25.5%) patients who underwent unilateral MAST received postoperative RT. All patients underwent axillary surgery, with 255 (65.1%) patients undergoing sentinel lymph node biopsy only and 137 (34.9%) patients undergoing axillary lymph node dissection. 61 (15.6%) patients reported having lymphedema.

### BREAST-Q results by breast surgery strategy

**Figure 1:**
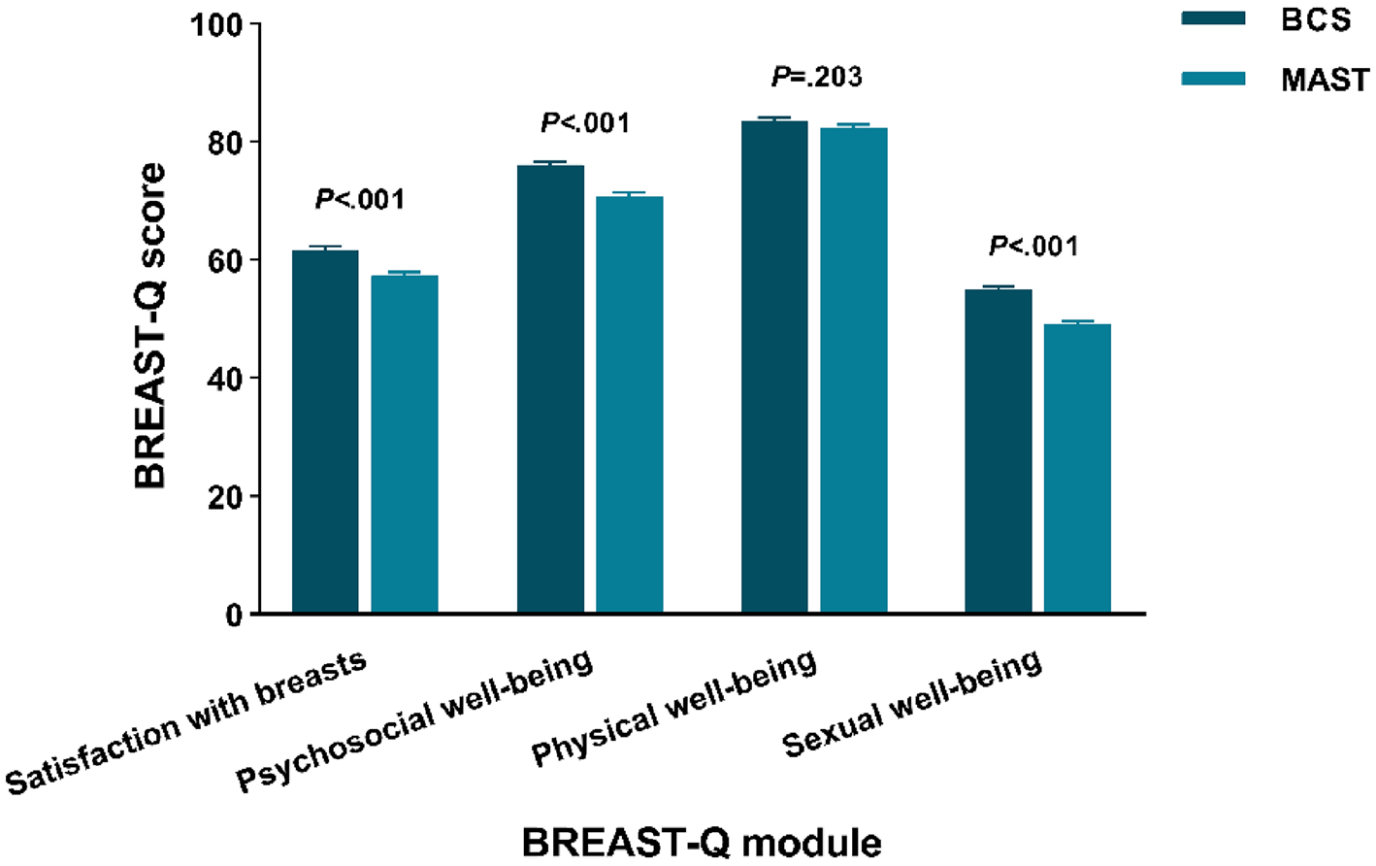
Unadjusted BREAST-Q mean scores by breast surgery strategy. BCS: breast-conserving surgery; MAST: mastectomy.

Figure 1 illustrates unadjusted mean BREAST-Q scores by breast surgery strategy. Satisfaction with breasts, psychosocial well-being and sexual well-being were significantly different among the groups (*P* < 0.001). BCS group showed higher scores in satisfaction with breasts (61.70), psychosocial well-being (76.01), physical well-being (83.52) and sexual well-being (55.06), while the scores for MAST group is lower (satisfaction with breasts: 57.30, psychosocial well-being: 70.83, physical well-being: 82.40 and sexual well-being: 49.21).

### Satisfaction with breasts

**Figure 2:**
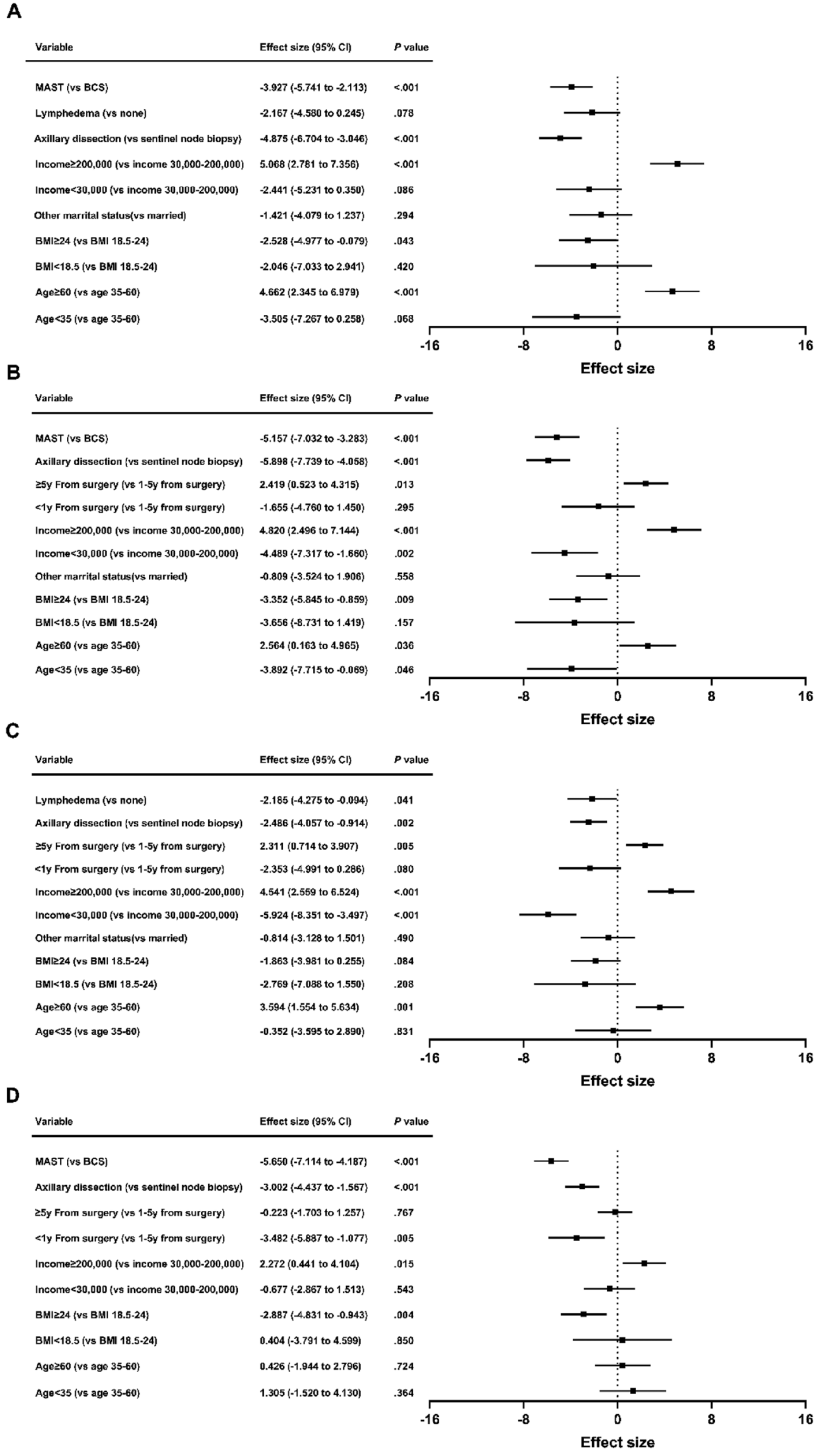
Patient and treatment factors associated with breast satisfaction (**A**), psychosocial well-being (**B**), physical well-being (**C**) and sexual well-being (**D**) scores by breast surgery strategy. MAST: mastectomy; BCS: breast-conserving surgery; BMI: body mass index; CI: confidence interval.

Higher scores in satisfaction with breasts correlated independently with age ≥ 60 (β = 4.662; 95% CI = 2.345 to 6.979; *P* < 0.001) and patient-reported income ≥ 200,000 (β = 5.068; 95% CI = 2.781 to 7.356; *P* < 0.001). Lower scores were associated with BMI ≥ 24 (β = − 2.528; 95% CI = − 4.977 to − 0.079; *P* = 0.043), axillary dissection (β = − 4.875; 95% CI = − 6.704 to − 3.046; *P* < 0.001) and MAST (β = − 3.927; 95% CI = − 5.741 to − 2.113; *P* < 0.001) (Fig. 2A). Patient-reported income < 30,000 and lymphedema showed significance only in univariate analysis. Other factors exhibited no significant association.

### Psychosocial well-being

Better psychosocial well-being correlated with age ≥ 60 (β = 2.564; 95% CI = 0.163 to 4.965; *P* = 0.036), patient-reported income ≥ 200,000 (β = 4.820; 95% CI = 2.496 to 7.144; *P* < 0.001), and ≥ 5y from surgery (β = 2.419; 95% CI = 0.523 to 4.315; *P* = 0.013). Poor psychosocial well-being was linked to age < 35 (β = − 3.892; 95% CI = − 7.715 to − 0.069; *P* = 0.046), BMI ≥ 24 (β = − 3.352; 95% CI = − 5.845 to − 0.859; *P* = 0.009), patient-reported income < 30,000 (β = − 4.489; 95% CI = − 7.317 to − 1.660; *P* = 0.002), axillary dissection (β = − 5.898; 95% CI = − 7.739 to − 4.058; *P* < 0.001) and MAST (β = − 5.157; 95% CI = − 7.032 to − 3.283; *P* < 0.001) (Fig. 2B). Chemotherapy was only significant in univariate analysis. Other variables showed no significant association.

### Physical well-being

Factors associated with better physical well-being were age ≥ 60 (β = 3.594; 95% CI = 1.554 to 5.634; *P* = 0.001), patient-reported income ≥ 200,000 (β = 4.541; 95% CI = 2.559 to 6.524; *P* < 0.001), and ≥ 5y from surgery (β = 2.311; 95% CI = 0.714 to 3.907; *P* = 0.005). Conversely, patient-reported income < 30,000 (β = − 5.924; 95% CI = − 8.351 to − 3.497; *P* < 0.001), axillary dissection (β = − 2.486; 95% CI = − 4.057 to − 0.914; *P* = 0.002) and lymphedema (β = − 2.185; 95% CI = − 4.275 to − 0.094; *P* = 0.041) were associated with poorer physical well-being (Fig. 2C). < 1y from surgery was only significant in univariate analysis. Other factors lacked significant association.

### Sexual well-being

Multivariate analysis indicated lower sexual well-being scores with BMI ≥ 24 (β = − 2.887; 95% CI = − 4.831 to − 0.943; *P* = 0.004), < 1y from surgery (β = − 3.482; 95% CI = − 5.887 to − 1.077; *P* = 0.005), axillary dissection (β = − 3.002; 95% CI = − 4.437 to − 1.567; *P* < 0.001), and MAST (β = − 5.650; 95% CI = − 7.114 to − 4.187; *P* < 0.001). Patient-reported income ≥ 200,000 (β = 2.272; 95% CI = 0.441 to 4.104; *P* = 0.015) correlated with elevated sexual well-being (Fig. 2D). Lymphedema was significant in univariate analysis. Other variables exhibited no significant correlation.

### BREAST-Q results by local therapy strategy

**Figure 3:**
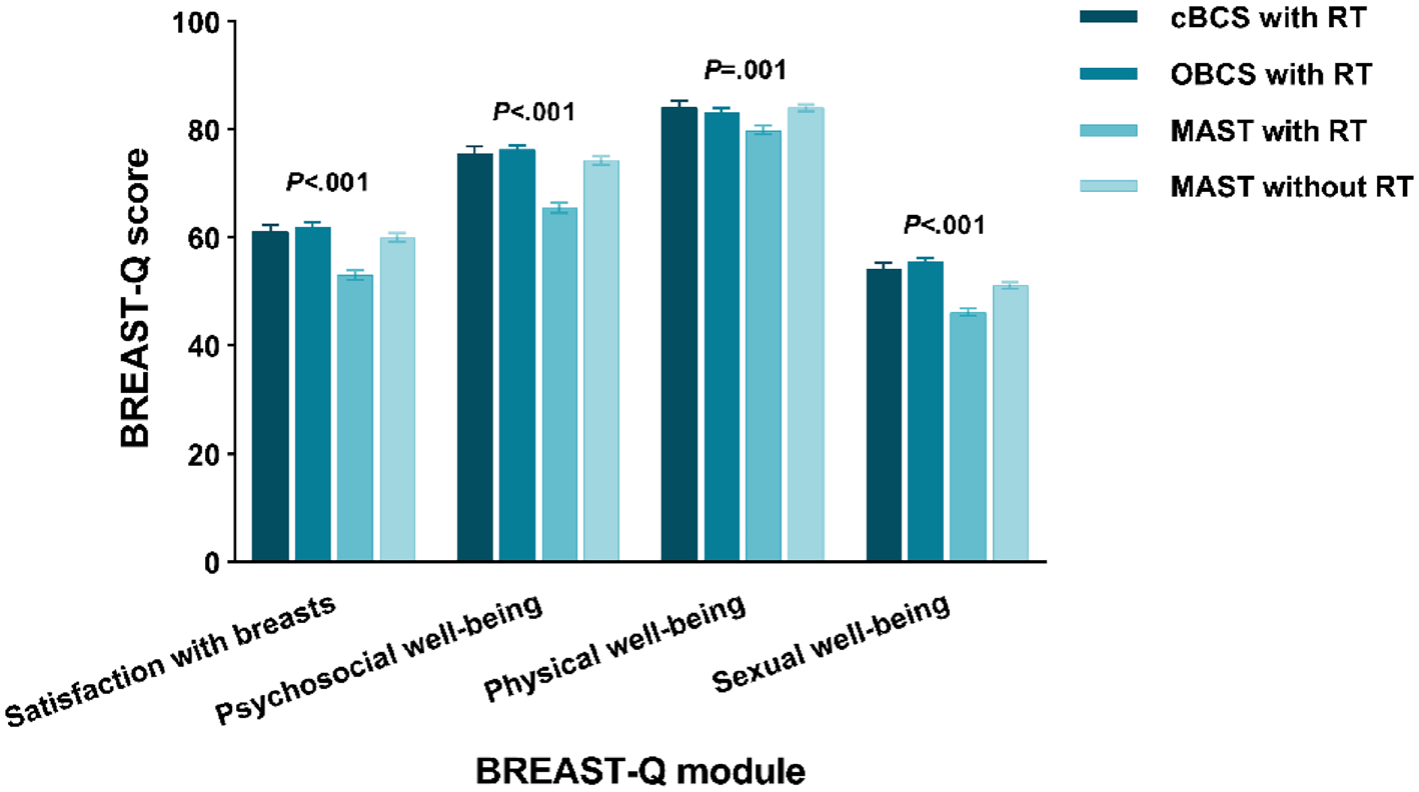
Unadjusted BREAST-Q mean scores by local therapy strategy. RT: radiotherapy; cBCS: conventional breast-conserving surgery; OBCS: oncoplastic breast-conserving surgery; MAST: mastectomy.

To assess if there were enhancements in quality of life among women who underwent OBCS, we performed similar analyses among the subgroups. Figure 3 illustrates unadjusted mean BREAST-Q scores by local therapy strategy. All four domains were significantly different (*P* < 0.05). OBCS with RT group showed highest scores in satisfaction with breasts (61.99), psychosocial well-being (76.27) and sexual well-being (55.53). cBCS with RT group yielded the highest physical well-being score (84.10). The lowest domain scores were in MAST with RT group (satisfaction with breasts: 53.11, psychosocial well-being: 65.49, physical well-being: 79.89 and sexual well-being: 46.24).

**Figure 4:**
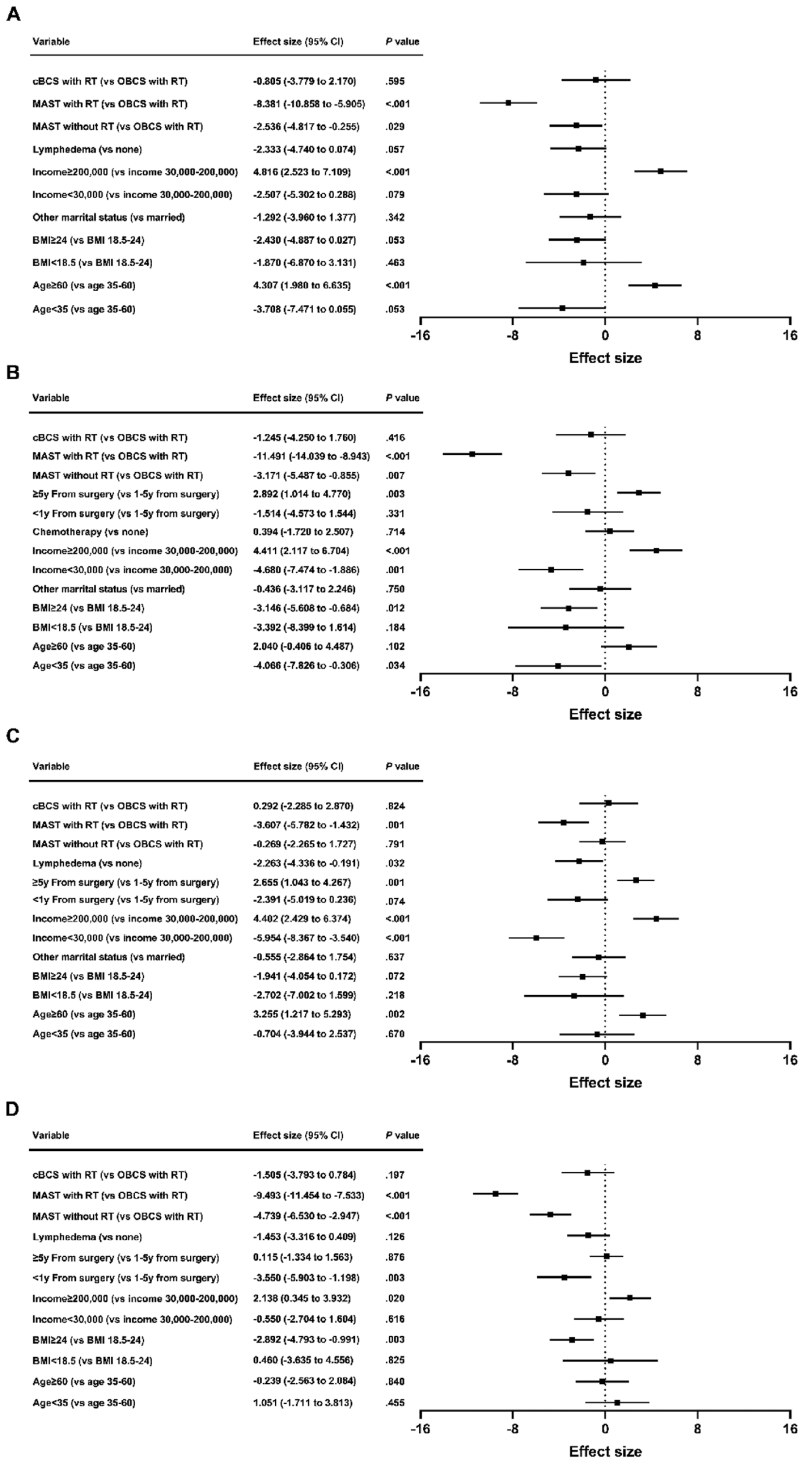
Patient and treatment factors associated with breast satisfaction (**A**), psychosocial well-being (**B**), physical well-being (**C**) and sexual well-being (**D**) scores by local therapy strategy. cBCS: conventional breast-conserving surgery; OBCS: oncoplastic breast-conserving surgery; MAST: mastectomy; RT: radiotherapy; BMI: body mass index; CI: confidence interval.

Multivariate analysis indicated that MAST with RT was associated with poor breast satisfaction (β = − 8.381; 95% CI = − 10.858 to − 5.905; *P* < 0.001), psychosocial well-being (β = − 11.491; 95% CI = − 14.039 to − 8.943; *P* < 0.001), physical well-being (β = − 3.607; 95% CI = − 5.782 to − 1.432; *P* = 0.001) and sexual well-being (β = − 9.493; 95% CI = − 11.454 to − 7.533; *P* < 0.001). MAST without RT was associated with decreased breast satisfaction (β = − 2.536; 95% CI = − 4.817 to − 0.255; *P* = 0.029), psychosocial well-being (β = − 3.171; 95% CI = − 5.487 to − 0.855; *P* = 0.007) and sexual well-being (β = − 4.739; 95% CI = − 6.530 to − 2.947; *P* < 0.001). cBCS with RT was not associated with BREAST-Q scores on univariate or multivariate analysis. The statistically significant factors correlated with BREAST-Q scores were mostly consistent with the outcomes of the breast surgery models (Fig. 4).

## Discussion

The rates of BCS and breast reconstruction after mastectomy are significantly lower in China than in Western countries^15^. One contributing factor is that Chinese women typically have smaller breast sizes than women in Western countries, while presenting with larger breast tumor volumes at the time of initial diagnosis, making BCS challenging. Additionally, some Chinese patients adhere to outdated beliefs and have concerns about potential impacts on treatment outcomes or cancer recurrence associated with BCS. OBCS provides acceptable long-term oncological outcomes and has extended treatment options for patients who would traditionally be candidates for mastectomies^6^. In recent years, there has been a clear change in the emphasis of surgical oncology in China, with a growing emphasis on utilizing modern oncoplastic surgical techniques to perform more breast conserving surgeries. Given the increasing prevalence of OBCS, it is essential to examine its impact on quality of life.

In this single-center prospective study, discernible disparities in quality of life surfaced among patients with breast cancer undergoing various local treatment strategies within ten years of surgery. Patients opting for more extensive surgery, particularly when combined with RT, experienced diminished quality of life; satisfaction with breasts; and psychosocial, physical, and sexual well-being. This aligns with findings from prior studies. Engel et al.’s study^16^ has shown that patients undergoing BCS reports a higher quality of life compared to those opting for mastectomy. This improvement is often linked to the conservation of the breast and the associated psychological advantages. BCS enables breast conservation, leading to enhanced body image and self-esteem. Patients undergoing BCS may experience less psychological distress and enjoy better psychosocial well-being due to breast conservation. Additionally, BCS has a lesser impact on sexual well-being in comparison to mastectomy, as it retains natural breast tissue.

This study’s findings concur with those of Otsuka et al.’s study^17^ in that oncoplastic surgery improved satisfaction with breasts. However, in Otsuka et al.’s study, the quality of life score was not elevated by OBCS (major breast surgery: 154.5 ± 24.6; minor breast surgery: 159.0 ± 20.8; OBCS: 158.7 ± 14.0). Although differences exist between major breast surgery and OBCS, the difference is not pronounced. In the present study, psychosocial and sexual well-being scores were elevated compared to MAST. Additionally, patients who underwent OBCS had better physical well-being scores than those who underwent MAST with RT and equal physical well-being scores than those who underwent MAST without RT. This may be attributable to the omission of RT, reduced chemotherapy and lymphedema in the MAST without RT group. Previous studies^18,19^ have highlighted RT, chemotherapy, and lymphedema as adverse determinants of quality of life.

Rose et al.^20^ suggested that patients who underwent OBCS showed significant improvement in the “psychosocial well-being” module compared to cBCS, while no significant differences were observed between the two groups in the “physical health,” “breast satisfaction,” and “sexual health” modules. Furthermore, a meta-analysis^21^ indicated improved quality of life with OBCS compared with cBCS in patients with early-stage breast cancer, with better physical and psychological well-being, higher self-esteem, and a more stable body image, leading to improved social and emotional functioning. However, the clinical studies included in the meta-analysis were predominantly small- sample studies from single centers, and the surgical approaches varied. This study identified no significant differences in any of the quality of life modules between the patients who underwent OBCS and those who underwent cBCS, which is consistent with the findings of de Oliveira-Junior et al^22^. This may be because the present study’s follow-up time was longer, and several aspects of OBCS will decline over time^23^. In our study, the tumor lesion on imaging before surgery averaged 2.11 ± 0.67 cm in OBCS subgroup, and 1.62 ± 0.52 cm in cBCS subgroup. Smaller lesions are more likely to undergo cBCS, resulting in comparable cosmetic outcomes between the two surgical groups. Moreover, the limited number of BCS patients in our study is a significant factor that limits the ability to detect differences in quality of life between OBCS and cBCS subgroups.

In addition to the type of surgery, other clinical factors such as BMI (≥ 24), income (< 30,000), < 1y from surgery, axillary dissection, and lymphedema were negatively correlated with quality of life. Identifying these risk factors can facilitate early postoperative intervention and ultimately improve the postoperative quality of life of patients with breast cancer. Age (≥ 60) and ≥ 5y from surgery were associated with enhanced quality of life. Breast cancer patients can experience significant effects from the disease itself and the ongoing adjuvant therapies, both after diagnosis and during the treatment process^24^. These are all factors that lead to decreased quality of life within 5 years, especially within 1 year, rather than ≥ 5y after surgery. Moreover, good economic status was associated with better satisfaction with breasts, and psychosocial, physical, and sexual well-being. Patients with improved financial circumstances can access higher-quality healthcare services, opt for more expensive treatment options that may improve aesthetic outcomes. The financial advantage also affords patients more opportunities for supportive care, counseling, and resources to manage the challenges of breast cancer treatment and recovery, resulting in a decrease in stress, anxiety, and depression. These enhancements can have a positive impact on patients’ self-perception, confidence, and overall satisfaction with their breast appearance, all of which are closely connected to sexual health and intimacy. Notably, other studies^25,26^ found an association between economic status and quality of life.

This study has some limitations. It was a cross-sectional, single-time, survey-based prospective study; therefore, the baseline quality of life of patients before surgery was not recorded, which may have influenced their choice of surgical approach and postoperative quality of life. Additionally, this study did not identify patients who chose MAST due to refusal of BCS; patients who selected MAST based on personal preferences may have different quality-of-life scores. Furthermore, this study did not include patients with postmastectomy breast reconstructions, which may improve quality of life of postmastectomy patients. Finally, given that this was a single-center small-sample study, studies with larger sample sizes are required to further confirm the findings of this study. Nevertheless, patient-reported questionnaires can provide basic information on quality of life and assist in identifying potential areas requiring intervention during the patient’s survival period.

## Conclusion

OBCS is an acceptable option for patients with larger tumors who are not suitable for cBCS because it allows them to conserve their breasts^6^. This study demonstrated that patients who had their breast conserved reported a higher quality of life compared to mastectomy patients. Despite extensive incisions, additional tissue manipulation, and potential flap reconstruction, patients who underwent OBCS did not report a lower quality of life than those who underwent cBCS. Furthermore, they experienced significantly enhanced quality of life compared with patients who underwent MAST, particularly in the domains of satisfaction with breasts, psychosocial well-being, and sexual well-being. Quality of life data should be incorporated into decision support tools to assist patients with breast cancer in selecting the surgical approach, and discussions with patients should include information regarding quality of life to ensure that they understand the long-term impacts of different surgical approaches. This is particularly crucial because most patients with breast cancer have an extended postoperative survival period. Our data can support further improvements in Chinese breast surgical care for better survival and quality of life.

## Data Availability

The datasets generated and/or analyzed during the current study are not publicly available due to Chinese law but are available from the corresponding author on reasonable request.
